# Characterization of the complete mitochondrial genome of blacktip shark *Carcharhinus limbatus* (Carcharhiniformes: Carcharhinidae)

**DOI:** 10.1080/23802359.2021.1914214

**Published:** 2022-02-15

**Authors:** Xiaolin Huang, Zanhu Zhou, Tinghe Lai, Binyuan He, Demin Zhang

**Affiliations:** a School of Marine Sciences, Ningbo University, Ningbo, China; b Zhejiang Mariculture Research Institute, Wenzhou, China; c Xiamen Customs, Xiamen, China; d Guangxi Academy of Oceanography, Nanning, China

**Keywords:** Mitochondrial genome, *Carcharhinus limbatus*, phylogenetic position

## Abstract

In this study, we aimed to determine the complete mitochondrial genome of blacktip shark *Carcharhinus limbatus*. The mitochondrial genome was 16,705 bp in length, including 13 protein-coding genes, 22 tRNA genes, 2 rRNA genes, and a control region. Phylogenetic analysis was done using the Bayesian inference method, which showed a close relationship between *C. limbatus* and *C. amblyrhynchoides*.

*Carcharhinus limbatus* (family: *Carcharhinidae*) is globally distributed across the coastal tropical and subtropical waters. It was first described in Johannes Muller’s book Systematische Beschreihung der Plagiostomen (Muller and Henle 1841). *C. limbatus* is commonly known as the blacktip shark due to the black edges of the fins (Compagno 1984). It can also be distinguished based on its stout, fusiform body with a pointed snout, long gill slits, and no ridge between the dorsal fins. This species is important to both commercial and recreational fisheries. The International Union for Conservation of Nature (IUCN) has identified this species as near threatened based on its low reproductive rate and high value to fisheries (Musick and Fowler 2000). This is the first study that determined the complete mitochondrial genome and the phylogenetic position of the *C. limbatus*.

A specimen of *C. limbatus* was collected from the fishing pier in Ningbo, Zhejiang Province of China (geographic location: 29°11′33.9″ N, 121°54′57.7″ E). The specimen was preserved in 95% ethanol and deposited at Marine Biology Museum of Zhejiang Mariculture Research Institute (http://www.zjmri.com.cn/, Xiaolin Huang, xiaolinnlh@hotmail.com), with the collection number of NUCLSZK170214. All animal experimental protocols were approved by the guidelines of the Animal Research and Ethics Committees of NBU. The DNA was extracted from the muscle of *C. limbatus*, followed by LA-PCR using the Takara TMLA-Taq DNA polymerase kit. Primers were designed for each long-range PCR. All samples were purified using the gel purification kit (Invitrogen) after recovering them from 1.5% TBE agarose gel. The purified PCR products were sequenced on an ABI 3730 automated sequencer with ABI PRISM BigDye Terminators v3.0 Cycle Sequencing (ABI) via the primer-walking strategy. The detailed extraction and sequencing methods followed the procedure of Wang et al. (Wang et al. 2020).

The complete mitogenome was annotated using the software of Sequin v16.0 (National Library of Medicine, Bethesda, MD, USA) and it was aligned against mitogenomes from other Carcharhinid species using DNAMAN. The results of tRNAscan-SE 2.0 (Lowe and Chan 2016) in the default search mode showed that mitochondrial tRNA genes were folded into typical secondary structures. Twelve protein-coding genes (except *ND6*) and two rRNA genes were aligned in batches using MAFFT (Katoh and Standley 2013) plugin integrated into PhyloSuite (Zhang et al. 2020). Then, we concatenated the first and second codons of the 12 protein-coding genes and 2 rRNA genes as a dataset. We used PartitionFinder2 to search for the best model of the three pre-defined data blocks. According to BIC, GTR + I + G was selected as the optimal model for all three blocks. Next, gene trees were constructed in Bayesian inference frameworks to assess the phylogenetic position of *C. limbatus*. The analysis was performed using 16 *Carcharhinidae* species and *Triaenodon obesus* was set as the outgroup, for whom complete mitogenomes were available in the GenBank. Bayesian tree was estimated using MrBayes 3.2.6 (Ronquist et al. 2012) under the partition model (2 parallel runs, 10000 generations), where the initial 25% of sampled data were discarded as burn-in.

The complete mitochondrial genome of *C. limbatus* comprised 16,705 bp (Genbank accession: MW026667), including 13 protein-coding genes, 2 tRNAs, 22 tRNAs, and a noncoding region. The base composition of the genomes was as follows: A (31.39%), T (30.28%), C (25.13%), and G (13.21%), which demonstrated the A + T-rich (61.67%) feature. Most of the PCGs and tRNA genes were encoded on the H-strand. Only *ND6* and eight tRNA genes (tRNA^Gln^, tRNA^Ala^, tRNA^Asn^, tRNA^Cys^, tRNA^Tyr^, tRNA^Ser^, tRNA^Glu^, and tRNA^Pro^) were encoded on the L-strand. The length of the 13 protein-coding genes ranged from 168 bp (ATP8) to 1830 bp (*ND5*). The 12 PCGs had conventional ATG and GTG as the initiation codons, while *ND6* had CCT as the initiation codon. Ten PCGs ended with conventional terminal codons (TAA/TAG), while *ND4*, *ND6* and CO II had an incomplete stop codon T. The large ribosomal gene (16S) was 1670 bp long and was located between tRNA^Val^ and tRNA^Leu^; the small (12S) was 957 bp long and was located between tRNA^Phe^ and tRNA^Val^. *C. limbatus* contained a complete set of 22 tRNAs individually ranging from 68 to 75 bp in length. The noncoding control region was 1, 067 bp long, and was located between tRNA^Phe^ and tRNA^Pro^.

The results of Bayesian analysis showed a topology with strong posterior probability values, suggesting that the phylogenetic tree was well-supported. The topology of the phylogenetic tree was consistent with the results of previous studies, which confirmed the basic relationships in the *Carcharhinus* genus (Johri et al. 2020). *Carcharhinus* involves four main groups, and the tree showed that the most closely related species of *C. limbatus* was *C. amblyrhynchoides*, which formed a clade with *C. melanopterus*. However, the position of *C. macloti* + *C. tjutjot* clade differed from the results of Dunn et al. (Dunn et al. 2020). It clustered with a clade that included the other four Carcharhinus species. Due to the low statistical support, the relationship between these two clades could not be resolved in this study. *C. plumbeus* did not cluster with any species and showed a distant relationship with others. Thus, the discovery of more mitogenomes of *Carcharhinidae* species would enable a better understanding of the phylogenetic relationships among them.[Fig F0001]

**Figure 1. F0001:**
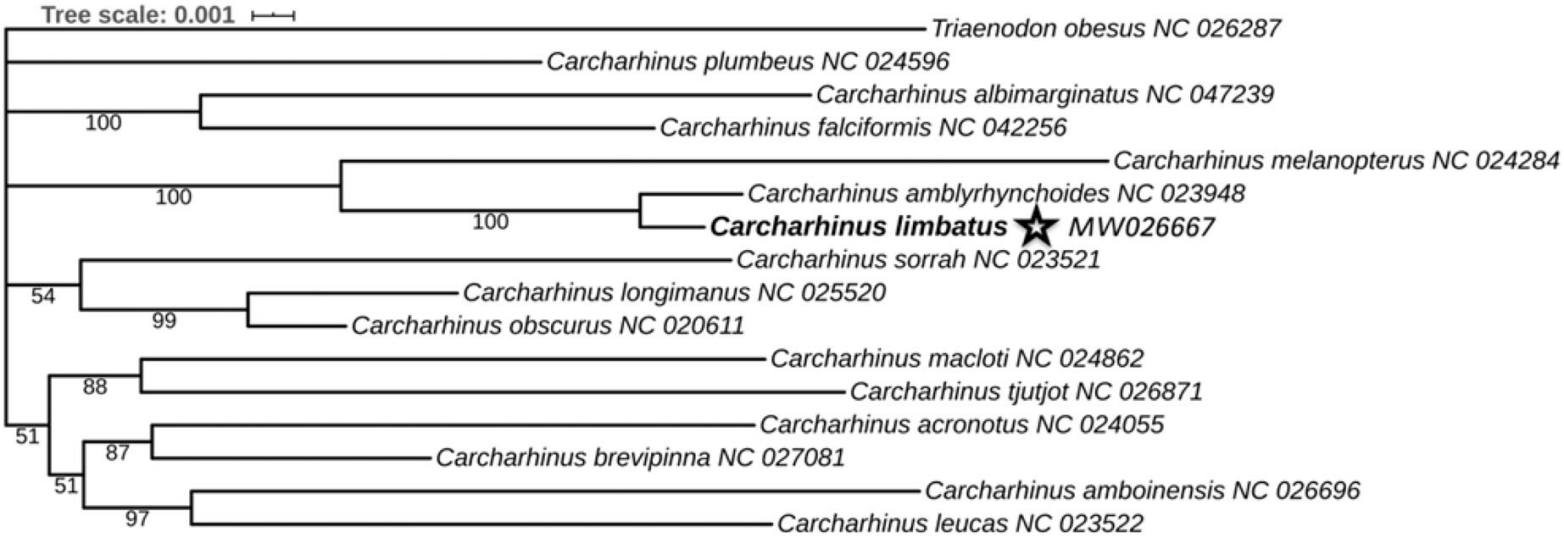
Phylogenetic position of Carcharhinus limbatus based on a comparison with the mitochondrial genome of 16 species. Bayesian posterior probability values are displayed next to the nodes. The asterisk denotes mitogenome of C. limbatus newly determined in this study.

## Data Availability

The genome sequence data that support the findings of this study are openly available in GenBank at https://www.ncbi.nlm.nih.gov/, accession number MW026667.

## References

[CIT0001] Compagno LJV. 1984. Sharks of the world: an annotated and illustrated catalogue of shark species known to date. Rome: Food and Agricultural Organization. p. 481–483.

[CIT0002] Dunn N, Johri S, Curnick D, Carbone C, Dinsdale EA, Chapple TK, Block BA, Savolainen V. 2020. Complete mitochondrial genome of the gray reef shark, *Carcharhinus amblyrhynchos* (Carcharhiniformes: Carcharhinidae). Mitochondrial DNA B Resour. 5(3):2080–2082. 33457750 10.1080/23802359.2020.1765208 PMC7782339

[CIT0003] Johri S, Chapple TK, Dinsdale EA, Schallert R, Block BA. 2020. Mitochondrial genome of the silky shark *Carcharhinus falciformis* from the British Indian Ocean Territory marine protected area. Mitochondrial DNA B Resour. 5(3):2416–2417. 33457810 10.1080/23802359.2020.1775147 PMC7782099

[CIT0004] Johri S, Dunn N, Chapple TK, Curnick D, Savolainen V, Dinsdale EA, and, Block BA. 2020. Mitochondrial genome of the Silvertip shark, *Carcharhinus albimarginatus*, from the British Indian Ocean Territory. Mitochondrial DNA B Resour. 5(3):2085–2086. 33457752 10.1080/23802359.2020.1765210 PMC7782225

[CIT0005] Katoh K, Standley DM. 2013. MAFFT multiple sequence alignment software version 7: improvements in performance and usability. Mol Biol Evol. 30(4):772–780. 23329690 10.1093/molbev/mst010 PMC3603318

[CIT0006] Lowe TM, Chan PP. 2016. tRNAscan-SE On-line: integrating search and context for analysis of transfer RNA genes. Nucleic Acids Res. 44(W1):W54–57. 27174935 10.1093/nar/gkw413 PMC4987944

[CIT0007] Muller JP, Henle F. 1841. Systematische Beschreibung der Plagiostomen. Berlin: Veit und comp.

[CIT0008] Musick JA, Fowler S. 2000. IUCN red list of threatened species: *Carcharhinus limbatus*. https://www.iucnredlist.org/en.

[CIT0009] Ronquist F, Teslenko M, van der Mark P, Ayres DL, Darling A, Höhna S, Larget B, Liu L, Suchard MA, Huelsenbeck JP. 2012. MrBayes 3.2: efficient Bayesian phylogenetic inference and model choice across a large model space. Syst Biol. 61(3):539–542. 22357727 10.1093/sysbio/sys029 PMC3329765

[CIT0010] Wang C, Chen H, Tian SL, Yang C, Chen X. 2020. Novel gene rearrangement and the complete mitochondrial genome of *Cynoglossus monopus*: insights into the envolution of the family Cynoglossidae (Pleuronectiformes). IJMS. 21(18):6895. 10.3390/ijms21186895 PMC7555148 32962212

[CIT0011] Zhang D, Gao F, Jakovlić I, Zou H, Zhang J, Li WX, Wang GT. 2020. PhyloSuite: an integrated and scalable desktop platform for streamlined molecular sequence data management and evolutionary phylogenetics studies. Mol Ecol Resour. 20(1):348–355. 31599058 10.1111/1755-0998.13096

